# How do questionnaire definitions of atopy status affect sample size calculations for asthma cohort studies in a population of Canadian children?

**DOI:** 10.1186/1710-1492-7-S2-A12

**Published:** 2011-11-14

**Authors:** Sarah Kwon, Elinor Simons, Teresa To, Sharon D Dell

**Affiliations:** 1 Hospital for Sick Children, Department of Pediatrics, Toronto, ON, Canada, M5G 1X8; 2 Hospital for Sick Children, Child Health Evaluative Sciences, Toronto, ON, Canada, M5G 1X8; 3 Hospital for Sick Children, Division of Respiratory Medicine, Toronto, ON, Canada, M5G 1X8

## Background

Skin prick tests (SPT) are the gold standard for determining atopy. In epidemiological studies of childhood allergy, questionnaire responses are often used to define atopy and predict sample size. Questionnaire-reported hayfever symptoms have shown 28-76% sensitivity and 21-94% specificity compared to SPT. We evaluated how questionnaire definitions of atopy affect sensitivity, specificity and sample size calculations in a population of Canadian children.

## Methods

We used questionnaire data from 5619 Toronto schoolchildren participating in the 2006 T-CHEQ study to determine 3 possible questionnaire definitions of atopy, including having any 1, any 2 or all 3 parent-reported physician diagnoses of hayfever, eczema or food allergy. In a nested case-control sample of 208 of these children, atopy was evaluated by SPT to14 common aeroallergens. Using SPT as the gold standard for atopy, we calculated sensitivity, specificity and sample size for a nested cohort study of particulate exposure and atopy outcome.

## Results

Compared with SPT, sensitivity, specificity and Youden’s index were 54.3%, 65.8% and 20.1% for 1 reported atopic condition and 24.4%, 98.7% and 23.1% for 2 reported atopic conditions, respectively (Table 1). Requiring at least 2 positive SPT for atopy did not change the sensitivity or specificity. Sample size calculations required 344 and 2948 participants for atopy defined by 1 or 2 atopic conditions, respectively.

**Table 1:** Sensitivity, specificity, PPV and NPV of questionnaire-based definitions of atopy compared to the gold standard of SPT and sample size calculations for a nested cohort study

Number of atopic conditions	Sensitivity	Specificity	Youden’s index	PPV	NPV	Sample size for nested cohort study
**1**	54.3%	65.8%	20.1%	72.6%	46.3%	344
**2**	24.4%	98.7%	23.1%	96.9%	44.8%	2948
**3**	4.7%	100.0%	4.7%	100.0%	39.3%	21034

## Conclusions

Questionnaire definitions of atopy in Canadian children have moderate sensitivity and specificity. More specific definitions decrease sensitivity and increase sample size requirement. Depending on the purpose of the proposed study, either definition of atopy may lead to an adequately-powered study.

